# Site-selective doping of ordered charge states in magnetite

**DOI:** 10.1038/s41467-020-15504-5

**Published:** 2020-04-03

**Authors:** E. Pachoud, J. Cumby, G. Perversi, J. P. Wright, J. P. Attfield

**Affiliations:** 1 Centre for Science at Extreme Conditions and School of Chemistry, University of Edinburgh, Edinburgh, EH9 3FD UK; 2 European Synchrotron Radiation Facility, 6 rue Jules Horowitz, BP220, 38043 Grenoble Cedex 9, France

**Keywords:** Solid-state chemistry, Electronic materials, Magnetic properties and materials, Electronic properties and materials

## Abstract

Charge ordering creates a spontaneous array of differently charged ions and is associated with electronic phenomena such as superconductivity, colossal magnetoresistances (CMR), and multiferroicity. Charge orders are usually suppressed by chemical doping and site selective doping of a charge ordered array has not previously been demonstrated. Here we show that selective oxidation of one out of eight distinct Fe^2+^ sites occurs within the complex Fe^2+^/Fe^3+^ ordered structure of 2%-doped magnetite (Fe_3_O_4_), while the rest of the charge and orbitally ordered network remains intact. This ‘charge order within a charge order’ is attributed to the relative instability of the trimeron distortion surrounding the selected site. Our discovery suggests that similar complex charge ordered arrays could be used to provide surface sites for selective redox reactions, or for storing information by doping specific sites.

## Abstract (web summary)

Charge ordering in magnetite is an important example of the complex behaviour that emerges in transition metal oxides. Here the authors show that doping causes selective oxidation of one site in the established trimeron pattern, introducing an additional charge-ordered structure.

## Introduction

Charge ordering is associated with notable electronic phenomena such as superconductivity in copper^1^ and bismuth^2^ oxides, CMR in manganese oxide perovskites^3^ and multiferroicity in iron oxides^4^. Charge orders are usually suppressed by doping although in a few cases such as CMR manganese oxides, the entire array rearranges into a new periodicity^5^. The concept of charge ordering was first proposed to account for the Verwey transition at *T*_V_ ≈ 125 K in magnetite^6,7^. The low-temperature order proved difficult to determine, but microcrystal X-ray diffraction has shown that the crystal structure changes from the cubic spinel type (space group $Fd\bar 3m$) to a complex monoclinic *Cc* superstructure below *T*_V_^8,9^. The Fe^2+^ sites within the Fe^2+^/Fe^3+^ charge-ordered array show a combination of orbital ordering and bonding to two Fe neighbours to form linear three-atom trimeron units, spin-polarised orbital molecules^10^. Orbital molecules associated with charge ordering have also been reported in other materials, such as CuIr_2_S_4_^11^, AlV_2_O_4_^12,13^, GaV_2_O_4_^14^ and CaFe_3_O_5_^15^. Complex charge orders may lead to spontaneous formation of structurally inequivalent cations of the same charge, for example, ordering of V^4+^/V^5+^ over eight inequivalent sites in α′-NaV_2_O_5_^16^, and notably in the *Cc* magnetite superstructure where charges are localised over sixteen distinct octahedrally coordinated sites, eight for Fe^2+^ and eight for Fe^3+^. Inequivalent cations of the same charge should, in principle, have slightly different redox potentials, but site selective doping of a charge ordered array has not previously been demonstrated.

Previous work has shown that magnetite can be hole-doped over a small range, up to *x* ≈ 0.035, with suppression of *T*_V_ and a change from sharp first order to broad second-order Verwey transitions reported around *x* = 0.012^17^. Equivalent effects were obtained for Fe-deficient samples Fe_3(1-δ)_O_4_ and Zn-doped materials Fe_3-*z*_Zn_*z*_O_4_, where the hole-doping *x* = 3δ = *z* corresponds to the number of holes introduced per Fe_3_O_4_ unit. Here, we show that highly selective doping is observed in a lightly oxidised magnetite microcrystal, where one of eight distinct Fe^2+^ sites is preferentially oxidised, despite the disorder associated with chemical doping.

## Results

### Structural studies of doped magnetite microcrystals

**Fig. 1:**
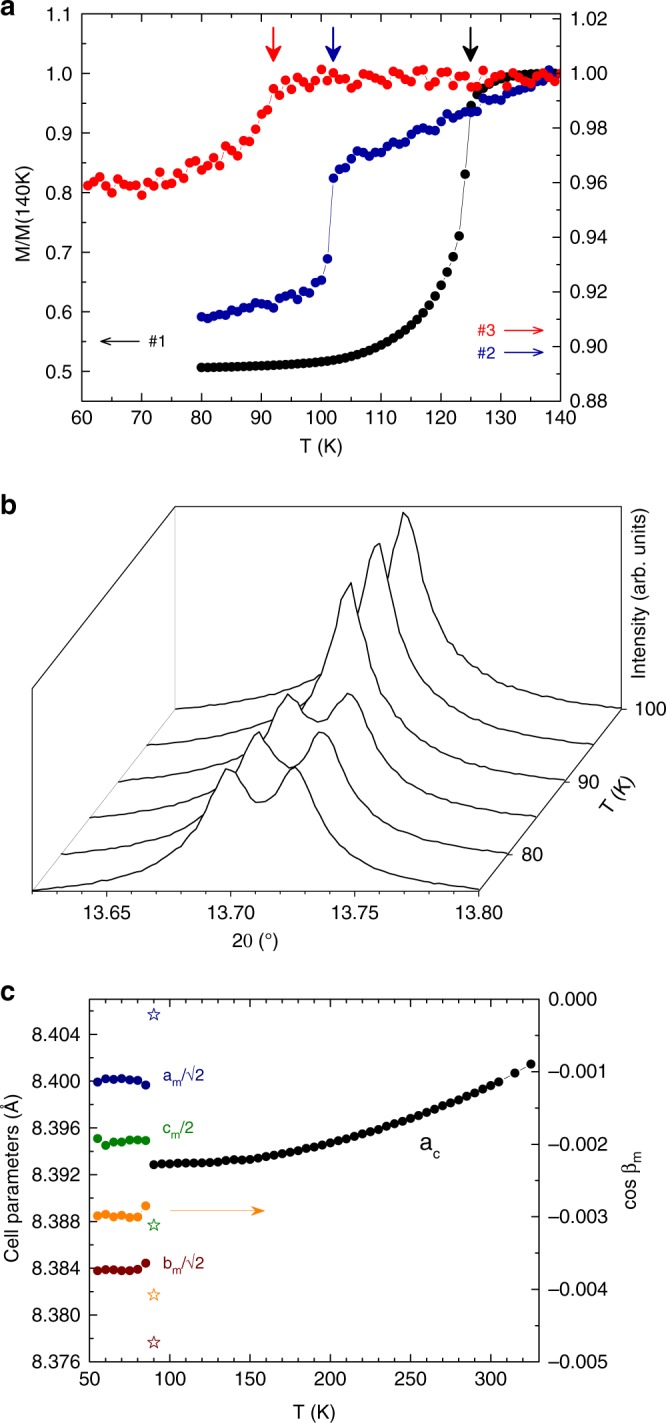
Characterisation of magnetite samples. **a** Zero-field cooled magnetisations of the powder for sample #1 and the microcrystals #2 and #3, normalised to 140 K values. The fields used were 100 Oe (#1 and #2) and 500 Oe (#3). The Verwey transition for each sample is indicated by a vertical arrow. **b** Low-temperature splitting of the cubic-(440) powder X-ray diffraction peak for sample #3, demonstrating that a lattice distortion occurs at the Verwey transition between 85 and 90 K. **c** Thermal evolution of unit-cell parameters derived from fits to powder X-ray profiles for #3 showing the monoclinic splitting at low temperatures. Values for sample #1 at 90 K^8^, shown as stars, demonstrate that the magnitude of the monoclinic distortion is greatly reduced by doping.

We compare results for three magnetite microcrystals. Details of the syntheses and magnetic and structural investigations are shown in “Methods”. Sample #1 is the highly stoichiometric magnetite (*x* = 0) previously used to determine the *Cc* superstructure^8^, and a sharp Verwey transition at 123 K is observed in the magnetisation measurements shown in Fig. 1. Sample #2 is an Fe-deficient Fe_3(1-δ)_O_4_ material that also has a sharp transition at *T*_V_ = 102 K, from which the estimated hole doping is *x* = 3δ = 0.0116. Zn^2+^ substitutes at tetrahedral cation sites that are not directly involved in the charge ordering, so a Fe_3-*z*_Zn_*z*_O_4_ sample was used to access higher hole-doping levels. This sample #3 shows a broad Verwey transition at *T*_V_ = 92 K from which doping *x* = *z* = 0.0228 is estimated.

Our samples #2 and #3, respectively, lie in the first- and second-order Verwey transition regimes. It was originally thought that second-order samples did not show a structural transition at *T*_V_, but a subsequent study reported that a distortion of the cubic cell is present in the second-order regime, modelled with a rhombohedral $R\bar 3m$ cell^18^. A recent Mössbauer and room temperature diffraction study suggested a change of lattice symmetry for Fe_3-*z*_Zn_*z*_O_4_ between first- and second-order regimes^19^, but no corresponding change in phonon spectra was found by inelastic neutron scattering^20^. A lattice distortion is clearly seen in the low-temperature powder diffraction patterns of sample #3 (Fig. 1b), and our microcrystal structural analysis shows that the same *Cc* supercell as for the undoped material is present. Hence, there is no change of low-temperature structural symmetry on passing from first- to second-order Verwey regimes. Instead, this anomaly may be attributed to the site-selective doping discovered below.

**Fig. 2:**
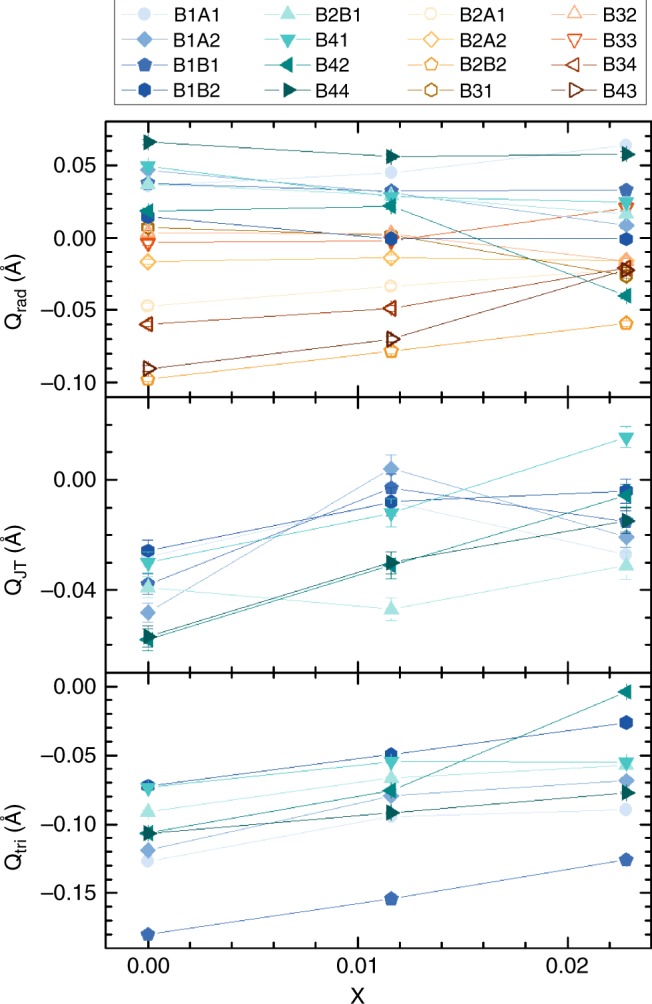
Evolution of local distortion modes with hole doping in magnetite. Plots from top to bottom show the *Q*_rad_ radial distortion parameters for all 16 octahedral B sites, the *Q*_JT_ tetragonal Jahn–Teller distortions for the eight Fe^2+^ sites, and the *Q*_tri_ trimeron shortening of Fe–Fe distances around the Fe^2+^ sites. B-site labels shown at the top are the same as used in previous studies^8,9^, with the eight Fe^2+^ sites in blue–green symbols to the left, and the eight Fe^3+^ sites with yellow–brown symbols to the right. Error bars are calculated from estimated standard deviations from the refinements.

**Fig. 3:**
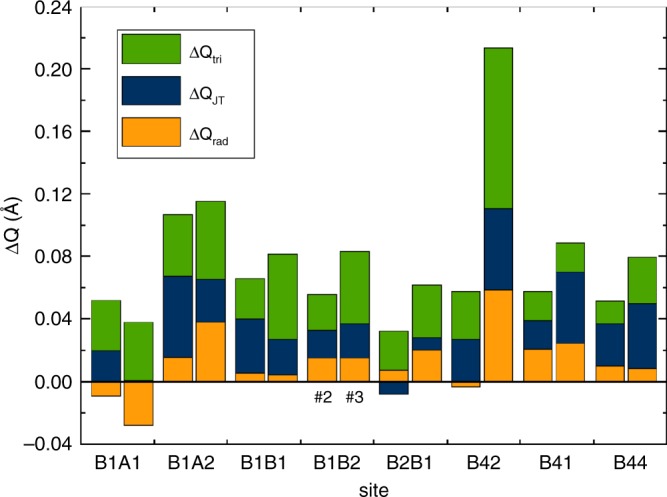
Changes of Fe^2+^ site distortion amplitudes on hole doping of magnetite. Changes in radial (−Δ*Q*_rad_), Jahn–Teller (Δ*Q*_JT_) and trimeron (Δ*Q*_tri_) modes for the eight distinct Fe^2+^ sites within the charge-ordered magnetite superstructure between the undoped sample #1 and samples #2 (left hand column for each site) or #3 (right hand columns) are shown. Negative values are shown below the horizontal axis.

The crystal structures of samples #2 and #3 below their Verwey transitions were determined from X-ray diffraction data using the same microcrystal method as for #1^8^. Both structures show the same qualitative charge ordering arrangement as for the *x* = 0 material. In order to quantify any small changes in the charge localisation, three local symmetry mode parameters are used, as defined in “Methods”. *Q*_rad_ measures the radial expansion of the FeO_6_ octahedra, equivalent to the average Fe–O distance, and hence is sensitive to the size difference between Fe^2+^ and Fe^3+^ cations. The tetragonal Jahn–Teller distortion due to the orbital order of high-spin 3d^6^ Fe^2+^ is given by *Q*_JT_, as defined in ref. ^6^. (Fe^3+^ is orbitally non-degenerate and so is not subject to Jahn–Teller distortion.) The shortening of the distances from each Fe^2+^ to its two neighbours in each trimeron is quantified by the *Q*_tri_ mode. Oxidation of an Fe^2+^ cation within the charge-ordered array is expected to lead to an decrease in *Q*_rad_ as Fe^3+^ is smaller than Fe^2+^, an increase in *Q*_JT_ due to loss of Fe^2+^ Jahn–Teller compressions that have negative *Q*_JT_ values, and an increase in *Q*_tri_ as shortened Fe–Fe distances are lost. Hence, −Δ*Q*_rad_, Δ*Q*_JT_ and Δ*Q*_tri_ are all expected to increase with hole doping of Fe^2+^ sites. Doping trends in these parameters mostly evolve smoothly with *x* as shown in Fig. 2. To display the combined changes for the 8 Fe^2+^ sites, the differences −Δ*Q*_rad_, Δ*Q*_JT_ and Δ*Q*_tri_ between values for undoped sample #1 and for the doped samples #2 and #3 are shown as a bar chart in Fig. 3. In total, 44 of the 48 Fe^2+^ Δ*Q* values are positive, demonstrating that the structure refinements are sensitive to the small changes induced by 1–2% hole doping. Most sites have values of the summed total distortion change Δ*Q*_tot_ = −Δ*Q*_rad_ + Δ*Q*_JT_ + Δ*Q*_tri_ in a common range between 0.01 and 0.12 Å, but it is striking that one site, B42 in crystal #3, has an outlying value of Δ*Q*_tot_ = 0.21 Å comprising large changes in all of the radial, Jahn–Teller and trimeron modes.

Chemical doping of magnetite through introduction of Fe-vacancies in sample #2 or Zn-substitution in sample #3 occurs at high temperatures, so the defects are located randomly through the lattice with respect to the charge order that emerges below *T*_V_. Hence, a statistically uniform oxidation of Fe^2+^ sites that happen to be adjacent to a defect is expected, and this accounts for the distribution of similar Δ*Q*_tot_ values for most of the Fe^2+^ sites in Fig. 3. However, it is evident that site B42 is substantially more oxidised in crystal #3 (site labels in the charge-ordered structure are shown on Fig. 3 of ref. ^9^), and this reveals a remarkable site-selectivity between the spontaneously created inequivalent Fe^2+^ sites within the charge-ordered magnetite structure. It is notable that this effect is seen at such a small hole-doping level. The *x* = 0.0228 doping of sample #3 is only around one-fifth of the *x* = 1/8 = 0.125 level needed to fully oxidise one of the eight distinct Fe^2+^ sites in the charge-ordered array, and so corresponds to oxidation on average of only 0.7 of the 32 Fe^2+^ sites in the monoclinic unit cell. No selective doping of B42 or any other site is evident for crystal #2 at the lower doping of *x* = 0.0116. Hence, it is likely that the previously reported changeover from first- to second-order Verwey transitions near *x* = 0.012 is due to the localisation of holes at the B42 site above this limit, although studies of further samples would be needed to confirm this. Long-range ordering of trimerons likely persists throughout the existence range of doped magnetites (up to *x* ≈ 0.035^17^), and robustness of disordered local trimeron distortions to high temperatures above *T*_V_ has also been reported recently^21^.

The low-temperature structure of magnetite was previously shown to consist of a network of corner-sharing trimerons, in which the extra *t*_2*g*_ electron of the central Fe^2+^ donor is significantly delocalised onto two adjacent B-site acceptors^8^. Seven of the eight structurally distinct trimerons have Fe^3+^ cations as the two acceptors, i.e., they have charge configuration Fe^3+^–Fe^2+^–Fe^3+^. However, the B42 site is unique in having a Fe^2+^–Fe^2+^(B42)–Fe^3+^ configuration, where another Fe^2+^ acts as a terminal charge acceptor. The present discovery that B42 is more easily oxidised that other Fe^2+^ sites can thus be attributed to the anomalous nature of the B42 trimeron. Electron–electron repulsion between the localised electrons of the adjacent Fe^2+^’s in this trimeron lowers the potential for ionisation of the B42 site relative to other Fe^2+^ ions.

## Discussion

**Fig. 4:**
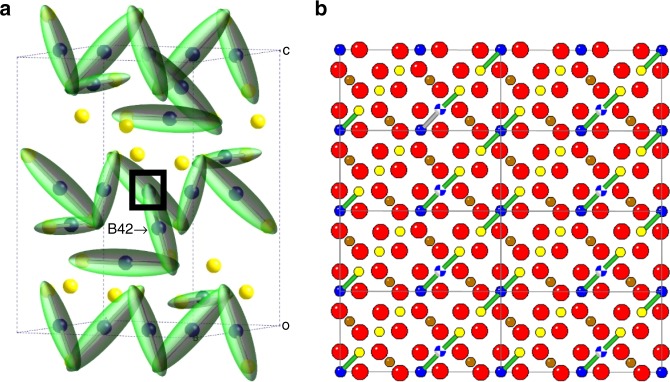
Charge and trimeron orders in magnetite. **a** Trimeron distribution in the low-temperature magnetite *Cc* cell, with Fe^2+^/Fe^3+^ shown as blue/yellow spheres and trimeron ellipsoids in green. The selectively oxidised B42 site is labelled, and the unusual termination of its trimeron by another Fe^2+^ is marked by a square. **b** (100) surface of cubic magnetite showing trimerons in the surface layer. The B42 sites (blue and white ‘beachballs’) are expected to be oxidised in preference to other trimerons. Charge-ordered Fe^2+^/Fe^3+^ are shown as blue/yellow spheres, tetrahedral Fe/O sites are brown/red.

The above results show that the complex spontaneous charge ordering in magnetite creates electron-localised Fe^2+^ sites of distinguishable redox activity. Although the charge-ordered superstructure of magnetite is very complex, accurate determination of the many variable structural parameters gives exquisite sensitivity to tiny changes in local charge states through the local radial, Jahn–Teller and trimeron-distortion modes. The discovered doping selectivity is remarkable, as it corresponds to a ‘charge order within a charge order’. It is also notable that the rest of the charge, orbital and trimeron network remains robust while the B42 sites are oxidised, at least up to the 2% doping in this study. This suggests that similar complex charge-ordered arrays could be used to provide surface sites for selective redox reactions, or for storing information by doping-specific sites. It is notable that the B42 trimerons lie parallel to the cubic-(100) plane of magnetite which can be grown as thin films, so it might be possible to access and directly measure the difference in ionisation potential between the B42 and other in-plane trimerons (Fig. 4b) using scanning nanoprobe microscopies.

In summary, this work demonstrates that it is possible to selectively dope one site within the spontaneously created charge-ordered array of magnetite without disrupting the rest of the electronically ordered network. This opens new possibilities to create a ‘charge order within a charge order’, and such materials could be used to provide surface sites for selective redox reactions, or for storing information by doping-specific sites.

## Methods

### Sample preparation

Sample #1 is a highly stoichiometric magnetite Fe_3(1-δ)_O_4_ with 3δ = 0.0000 originally provided by Prof. J. Honig that has been used in previously studies^8,9,21^. Samples #2 and #3 were synthesised through a modified route from Honig^17^. Small microcrystals of Fe_3(1-δ)_O_4_ (sample #2) or of Fe_3-*z*_Zn_*z*_O_4_ (sample #3) were obtained through firing Fe_2_O_3_ or an Fe_2_O_3_/ZnO mixture at 1580 °C under Ar flow. The stoichiometry of sample #2 Fe_3(1-δ)_O_4_^22^ was adjusted by reannealing the crystals in a vertical furnace with a fixed CO_2_/H_2_ buffer gas flow, to control the oxygen partial pressure according to the thermodynamic equilibrium of magnetite^17^ and reference tables derived from the JANAF database^23^.

### Magnetisation

Magnetic measurements were recorded with a Quantum Design SQUID magnetometer after zero-field cooling in fields of 100 Oe (samples #1 and #2) or 500 Oe (sample #3) during warming. Microcrystals were fixed in Eicosane within the gelatine capsule. Hole-doping levels *x* = 0.0116 and 0.0228 of samples #2 and #3, respectively, were estimated from their measured Verwey transition temperatures using the *T*_V_-*x* doping correlation plot in ref. ^17^.

### X-ray diffraction

Microcrystal X-ray diffraction data were collected on the diffractometer ID11 at ESRF (France). The wavelengths were all close to λ = 0.16 Å. The microcrystals (40–60 μm) were suspended in paratone oil in a litholoop. Cooling in a magnetic field from ambient temperature to 80–90 K (as described in ref. ^6^) was used to align the magnetic easy axis of the crystal with the field, thus helping to create a unique monoclinic *c*-axis below the Verwey transition. Temperature was controlled with a nitrogen cryostream. Data sets were recorded at 90 K (samples #1 and #2) or 80 K (sample #3), with varying omega-step sizes, exposure times, beam fluxes and at different phi settings, for high redundancy and to observe both low angle fundamental diffraction peaks and high-angle weak superstructure reflections.

Data reduction and integration for sample #2 were performed using the same procedure as for #1 in ref. ^8^, via Bruker SMART and SAINT software^24^. For crystal #3, data were indexed and integrated using CrysalisPro^25^ using an increased integration box (×2), and a filter for reflections above 50,000 counts to ensure detector linearity. Integrated results for each collection run were then processed using Sadabs^26^, applying a spherical absorption correction. The data were finally merged for refinement using Xprep^27^ to a maximum resolution of 0.28 Å. Structure refinements were carried out using SHELXL software^28^ and took both monoclinic and orthorhombic twin domains into account (excluding inversion twinning). Samples #2 and #3 were found to show substantial monoclinic twinning. All 168 of the variable atomic coordinates were refined independently, subject to the origin constraints of space group *Cc*.

High-resolution powder diffraction patterns for sample #3 were recorded at the ID22 beamline of ESRF from 55 to 325 K using a helium cryostat with *λ* = 0.354224 Å. Rietveld refinements were performed using Fullprof software^29^, and the monoclinic unit-cell parameters at 80 K were used as the values for the single-crystal refinement.

### Distortion modes

Local distortion mode parameters are used to quantify the magnitude and changes of local distortions of the low-temperature magnetite structure with doping. Distortions of FeO_6_ octahedra have been previously described by local modes *Q* in the O_h_ point group^8^.

The radial expansion (breathing) mode *Q*_rad_ is calculated from Eq. (1).

1 $$Q_{\mathrm{rad}} = \mathop {\sum}\limits_i {\frac{{d_i - \langle d\rangle }}{{\sqrt 6 }}}$$

where *d*_*i*_ are the six Fe–O distances in the octahedron and <*d*> is the global average octahedral Fe–O distance. This breathing mode describes changes in the cation size, and so is sensitive to charge ordering of large Fe^2+^ against small Fe^3+^.

The orbital ordering is characterised by the tetragonal Jahn–Teller mode *Q*_JT_, calculated as described in Supplementary Information to ref. ^8^.

A trimeron mode *Q*_tri_ sensitive to Fe–Fe distances is similarly defined for each Fe^2+^ in Eq. (2).

2 $$Q_{{\mathrm{tri}}} = \mathop {\sum}\limits_i {\frac{{BB_i - BB}}{{\sqrt 2 }}}$$

This is summed over the two Fe–Fe distances *BB*_*i*_ within the trimeron, where <*BB*> is the average of all nearest-neighbour B–B distances within the structure.

## Data Availability

Data that support the findings of this study have been deposited at 10.7488/ds/2783.
